# Successfully enucleation of a rare pancreatic schwannoma: A case report

**DOI:** 10.1016/j.ijscr.2025.110865

**Published:** 2025-01-13

**Authors:** Ben Abdessalem Abdelaziz, Yassine Kallel, Jbir Ichraf, Hazem Beji, Emna Chalbi, Hassen Touinsi

**Affiliations:** a Department of General Surgery, Hospital Mohamed Taher Maamouri, Nabeul, Tunisia; b Department of Anatomo Pathology, Hospital Mohamed Taher Maamouri, Nabeul, Tunisia

**Keywords:** Pancreatic schwannoma, Enucleation, Case report

## Abstract

**Introduction and importance:**

Pancreatic schwannoma (PS) is an extremely rare benign tumor also known as neurilemoma or neuroma. The majority of PS develop cystic lesions, and its preoperative diagnosis is challenging due to its tendency to mimic other lesions of the pancreas. Herein, we reported a case of body PS incidentally discovered in an 81-year-old male, which was successfully treated through enucleation.

**Case presentation:**

We report the case of a 81-year-old man. Who presented with a well-defined polycystic-tumor of about 2cm at the pancreatic body, overdrawn by a computed tomography scan, incidentally discovered. The patient underwent a laparotomy, and we performed an enucleation of the tumor successfully. Histopathological examination revealed spindle-shaped cells. Immunohistochemically studies showed S100-protein expression, confirmed a body pancreatic schwannoma. The postoperative course was uneventful.

**Clinical discussion:**

Schwannomas are rare mesenchymal-tumors, with PS accounting for only 1 % of cases. They are often difficult to diagnose as they mimic other pancreatic tumors. Imaging techniques like CT, MRI, and EUS-FNA aid in detection, but definitive diagnosis requires histological and immunohistochemical analysis. Treatment is typically enucleation for benign tumors, but larger or malignant tumors may need more extensive resections. Due to diagnostic challenges, aggressive surgeries are common. Prognosis is generally favorable, but regular follow-ups are recommended to monitor for recurrence.

**Conclusion:**

Although pancreatic schwannoma is rare, it should be included in the list of differential diagnoses of pancreatic masses, both solid and cystic. Both enucleation and radical surgical resections have revealed great therapeutic efficiency with a well prognosis without recurrences.

## Highlights

• Pancratic schwannomas are rare, benign tumors originating from Schwann cells, accounting for about 1% of all schwannomas.
• Only 97 cases have been reported in the past 50 years. They are often asymptomatic making preoperative diagnosis challenging.
• Treatment typically involves surgical enucleation, with a favorable prognosis.
• A case of an 81-year-old male with a pancreatic schwannoma was successfully treated through enucleation.
• Accurate preoperative diagnosis remains difficult despite imaging and EUS-FNA, with surgery as the primary treatment option.

## 1 Introduction

Schwannomas are the most prevalent form of peripheral nerve sheath tumors. They originate from Schwann cells responsible for safeguarding nerve cells within the nervous system [1]. These encapsulated tumors typically manifest in the head and neck regions, the trunk, as well as the flexor areas of both upper and lower extremities [2]. Although uncommon, these tumors can also occur within the pancreas representing approximately 1 % of all schwannomas [3,4]. The majority of schwannomas are benign. To the best of our knowledge, only 97 cases of pancreatic schwannomas have been documented in English literature within the past 50 years [5]. Most patients remain asymptomatic, with incidental detection of the tumor. In this report, we present a case of pancreatic schwannoma diagnosed in an 81-year-old male, which was successfully treated through enucleation.

This work has been reported in line with the SCARE 2023 criteria [6].

## 2 Case presentation

An 81-year-old male with a medical history of diabetes, sarcoidosis, and chronic respiratory failure was admitted to the pneumology department due to an acute respiratory failure caused by pneumonia. During his hospital stay, he experienced intermittent abdominal discomfort without weight loss, jaundice, or significant changes in appetite. A computed tomography (CT) scan of the chest was performed to evaluate his pneumonia and incidentally revealed a pancreatic mass. Subsequently, the patient was referred to our department for further evaluation.

**Fig. 1:**
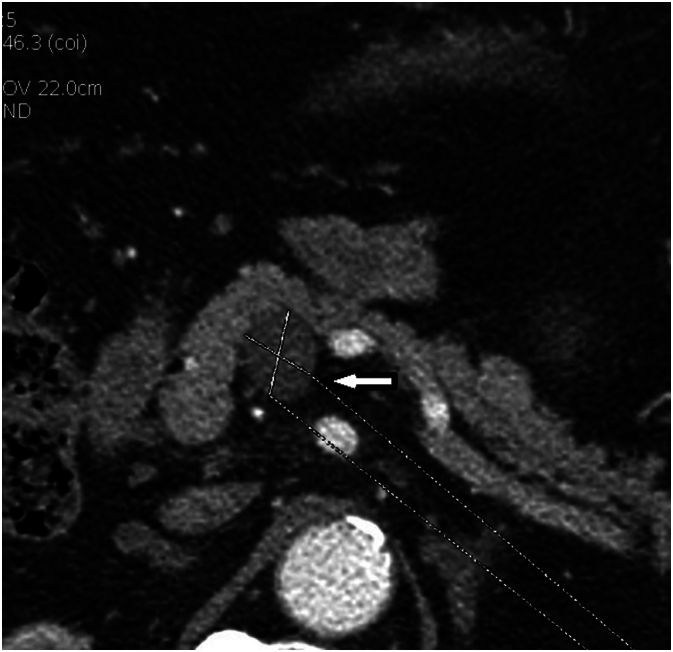
Post-contrast abdominal CT scan showing a well-defined hypodense lesion measuring 20 × 21 mm in the pancreatic body. The lesion is clearly delineated from the surrounding pancreatic parenchyma.

**Fig. 2:**
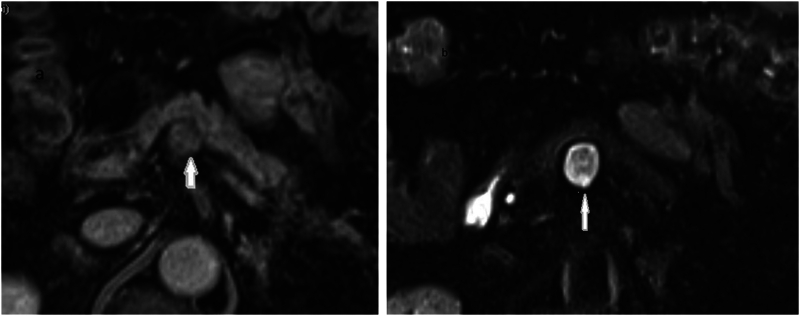
Pancreatic MRI. (a) Axial T1-weighted image (fat-suppressed, non-enhanced) showing a hypointense mass in the pancreatic body. (b) Axial T2-weighted image (fat-suppressed) depicting the lesion as heterogeneously hyperintense.

On admission, his physical examination was unremarkable, with no palpable abdominal masses or tenderness. The patient's medication history included metformin for diabetes and corticosteroids for sarcoidosis. Laboratory investigations revealed normal pancreatic enzyme levels (amylase and lipase) and no abnormalities in liver function tests. Tumor markers, including CA 19-9, were within normal limits. A chest X-ray showed resolving pneumonia, and an ECG indicated sinus rhythm without ischemic changes. Abdominal CT scan revealed a well-defined polycystic tumor measuring approximately 2 cm in the pancreatic body (Fig. 1). Pancreatic MRI depicted the mass as hypointense on T1-weighted images, heterogeneously hyperintense on T2-weighted images, and also hyperintense on diffusion-weighted imaging. Importantly, the mass exhibited no communication with the main pancreatic duct (Fig. 2). Based on these imaging findings and suspicion of a malignant lesion, the decision for surgical treatment was made. Preoperative preparation included routine blood tests, coagulation profile, and optimization of his respiratory function. Written informed consent for surgery was obtained.

The surgery was performed under general anesthesia via an open laparotomy approach with a transversal incision. Intraoperatively, we identified a 3 cm ovoid, well-circumscribed mass in the pancreatic body, enclosed by a fibrous capsule and with no contact with the splenic vessels. No enlarged lymph nodes or signs of lymphadenopathy were observed. The tumor was successfully enucleated without complications.

**Fig. 3:**
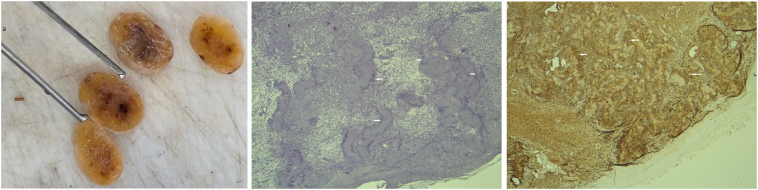
Histologic analysis of the pancreatic mass. (a) Macroscopic appearance of the tumor, showing a well-circumscribed, encapsulated lesion. (b) Histologic section (Hematoxylin and Eosin [H&E] stain) displaying spindle-shaped cells arranged in a palisading pattern without atypia. Abnormal cells are marked with arrows. Original magnification x100. (c) Immunohistochemistry analysis of the tumor, demonstrating strong and diffuse S-100 protein expression in the spindle cells (brown staining). Original magnification x4. Special staining highlights the cellular architecture and confirms the schwannoma diagnosis. Abnormal findings are indicated with arrows.

Histopathological examination revealed spindle-shaped cells arranged in a palisading pattern without atypia. Immunohistochemistry analysis displayed highly positive protein S-100 expression in the spindle cells, with negative results for CD34, CD117, KIT, DOG1, and smooth muscle actin (SMA) (Fig. 3), confirming the diagnosis of a pancreatic schwannoma.

The patient had an uneventful post-operative course and was discharged five days following surgery. Follow-up over 12 months showed no complications, recurrence, or evidence of malignancy.

## 3 Discussion

Herein, we reported a successful surgical treatment of a pancreatic schwannoma. The main strength of our work is the scarcity of this pathology. The main weakness is due to the non-performance of EUS with biopsies preoperatively.

Schwannomas, initially described by Verocay in 1910, represent mesenchymal neoplasms originating from Schwann cells lining peripheral nerve sheaths, notably devoid of neuroganglion cells [1]. Typically, these encapsulated tumors are observed in various anatomical regions, including the head and neck, trunk, and the flexor areas of both upper and lower extremities [2].

Despite their infrequency, pancreatic schwannomas constitute about 1 % of all reported cases of schwannomas [3,4]. The majority of schwannomas are benign. Diagnosing pancreatic schwannomas is challenging as they often mimic other pancreatic tumors clinically [4]. Typically benign and solitary, multiple malignant schwannomas can occur in patients with von Recklinghausen disease (VRD) [7]. These schwannomas, with a slight female predominance, are usually diagnosed at an average age of 55 [8].

Although pancreatic schwannomas may develop in any part of the pancreas, they are predominantly located in the pancreatic head [5]. Approximately one-third of patients remain asymptomatic, while common symptoms include abdominal pain, weight loss, and occasionally nausea, vomiting, dyspepsia, back pain, or the presence of a palpable mass [3]. Symptoms like anemia, melena, and jaundice are infrequent [3]. Laboratory tests, including tumor markers, generally fall within the normal range [9]. Imaging techniques such as CT, MRI, and endoscopic ultrasound aid in identifying pancreatic masses. However, distinguishing pancreatic schwannoma from other pancreatic tumors remains challenging due to its varied morphologies [10]. Consequently, these tumors often resemble other pancreatic tumors, leading to a high misdiagnosis rate. The most common differential diagnoses include cystic neoplasms (serous or mucinous), pancreatic neuroendocrine tumors, solid pseudopapillary tumors, mucinous cystadenocarcinomas, acinar cell carcinomas, and pancreatic pseudocysts.

Various image-guided aspirations, like endoscopic ultrasound-guided fine needle aspiration (EUS-FNA) or CT-guided FNA, allow for cytologic analysis of pancreatic tumors [11] and may aid in a more accurate preoperative diagnosis. However, the utilization of EUS-FNA for diagnosing pancreatic schwannomas is controversial due to a high incidence of false-negative findings and a diagnostic rate of only 52.9 % [9,11].

Definitive diagnosis relies on histological examination and immunohistochemical staining of a surgically resected specimen. Macroscopically, pancreatic schwannomas appear as well-circumscribed, encapsulated, homogeneous, yellow-tan nodules. Approximately two-thirds exhibit secondary degenerative changes such as cyst formation, calcification, hyalinization, hemorrhage, and xanthomatous infiltration [11]. Microscopically, these tumors feature organized hypercellular components (Antoni A areas) and hypocellular components with loose myxoid stroma (Antoni B areas). Strong positive immunohistochemical staining for S-100, vimentin, and CD-56 is typical, while negative staining occurs for cytokeratin AE1/AE3, CD34, CD117 (c-kit), desmin, and smooth muscle myosin [[11], [12], [13]].

Due to their benign nature, enucleation typically suffices for the majority of patients requiring intervention. However, large tumors with malignant behavior might necessitate an oncological margin-negative resection [14]. Pancreaticoduodenectomy was the most common surgical resection (33 %) followed by distal pancreatectomy with/without splenectomy (20 %), unspecified surgical resection (19 %), and enucleation (15 %) in a review of treatments by Zhang et al. [15].

The frequency of extensive radical resection may stem from challenges in obtaining accurate preoperative diagnoses of pancreatic schwannomas and differentiating them from other pancreatic neoplasms. Intra-operative histological diagnosis aids surgeons in selecting appropriate operative methods [15]. The decision on active surveillance, enucleation, or surgical resection should be individualized based on patient presentation, tumor size, location, laboratory studies, and histopathologic findings [11,15]. Following tumor excision Patients typically have a positive prognosis; however, due to uncertain risks of recurrence, regular imaging follow-ups are recommended [13,16].

### 3.1 Perspective

Future retrospective case series or systematic reviews can help study better this scarce pathology.

## 4 Conclusion

Achieving an accurate preoperative diagnosis of pancreatic schwannomas remains challenging despite utilizing various imaging methods. Surgery stands as the primary treatment for these tumors, with surgical approaches varying based on the tumor's location within the pancreas. Notably, an upsurge in accurately diagnosed cases through EUS-FNA has been observed recently, making surveillance an increasingly viable option. Following resection, patients with pancreatic schwannomas typically exhibit a favorable prognosis.

## Patient consent

Written informed consent was obtained from the patient for the publication of this case report and accompanying images. A copy of the written consent is available for review by the Editor-in-Chief of this journal on request.

## Ethical approval

Ethical approval is exempt/waived at our institution.

## Guarantor

Dr Ben Abdessalem Abdelaziz.

## Provenance and peer review

Not commissioned, externally peer-reviewed.

## Sources of funding

This research did not receive any specific grant from funding agencies in the public, commercial, or not-for-profit sectors.

## Research registration

N/A.

## Author contribution

Ben Abdessalem A, Kallel Y and Chalbi E contributed to manuscript writing and editing, and data collection.

Jbir I and Beji Hazem contributed to data analysis.

Touinsi H contributed to conceptualization and supervision; all authors have read and approved the final manuscript.

## Declaration of competing interest

No conflicts of interest.
